# Vestibular blueprint in early vertebrates

**DOI:** 10.3389/fncir.2013.00182

**Published:** 2013-11-19

**Authors:** Hans Straka, Robert Baker

**Affiliations:** ^1^Department Biology II, Ludwig-Maximilians-Universität MünchenPlanegg, Germany; ^2^Department of Neuroscience and Physiology, Langone Medical Center, New York UniversityNew York, NY, USA

**Keywords:** semicircular canal, otolith, eye movements, extraocular motoneurons, goldfish, hindbrain segment, vestibuloocular, vestibulospinal

## Abstract

Central vestibular neurons form identifiable subgroups within the boundaries of classically outlined octavolateral nuclei in primitive vertebrates that are distinct from those processing lateral line, electrosensory, and auditory signals. Each vestibular subgroup exhibits a particular morpho-physiological property that receives origin-specific sensory inputs from semicircular canal and otolith organs. Behaviorally characterized phenotypes send discrete axonal projections to extraocular, spinal, and cerebellar targets including other ipsi- and contralateral vestibular nuclei. The anatomical locations of vestibuloocular and vestibulospinal neurons correlate with genetically defined hindbrain compartments that are well conserved throughout vertebrate evolution though some variability exists in fossil and extant vertebrate species. The different vestibular subgroups exhibit a robust sensorimotor signal processing complemented with a high degree of vestibular and visual adaptive plasticity.

## INTRODUCTION

**FIGURE 1:**
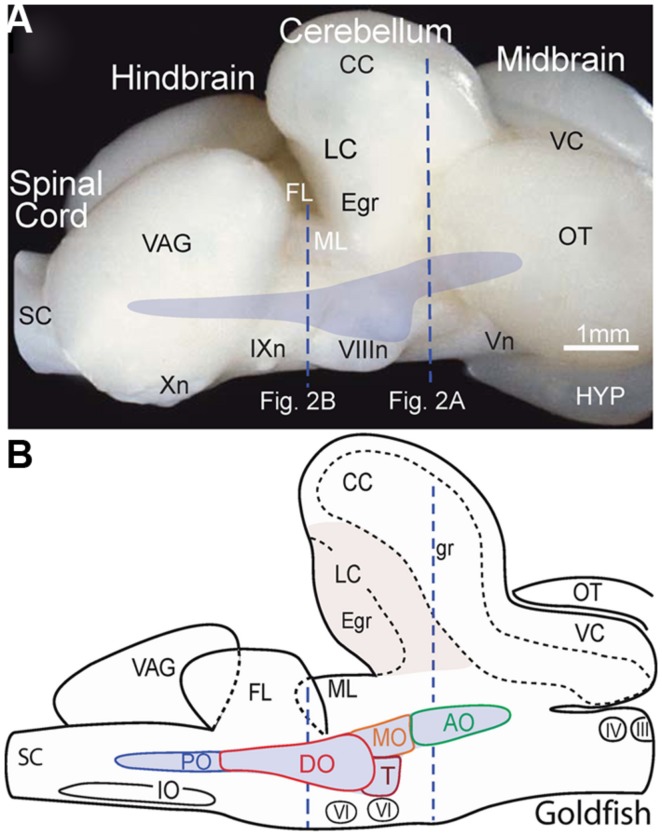
**Location of the octavolateral vestibular subdivisions within the goldfish hindbrain**. A side view of the intact brain **(A)** and sagittal schematic diagram **(B)** are drawn at the same scale. Vertical dashed lines indicate rostro-caudal levels of the coronal sections shown in **Figure 2**. AO, DO, MO, PO, anterior, descending medial, posterior octavolateral nuclei; CC, corpus cerebelli; Egr, external granule cell layer; FL, facial lobe; gr, corpus cerebelli granule cell layer; HYP, hypothalamus; III, IV, and VI oculomotor, trochlear, and abducens nuclei; VIIIn, IXn Xn, and Vn, vestibular, glossopharyngeal, vagal, and trigeminal nerves; IO, inferior olive; LC, lobus caudalis; ML, molecular layer of crus cerebelli; VC, valvula cerebelli; OT, optic tectum; SC, spinal cord; T, tangential nucleus; VAG, vagal lobe.

In the best-studied early vertebrates that largely represent fish species, particularly goldfish (*Carassius auratus*), the octavolateral nuclei occupy a considerable part of the dorso-lateral hindbrain between the fifth and tenth cranial nerves (**Figures 1A,B**). Functionally, the octavolateral nuclei are heterogeneous subgroups of neurons subserving four different sensory modalities: lateral line for detection of either water motion (mechanoreceptive) or objects (electroreceptive), auditory for acoustic pressure and vestibular for body motion (McCormick, 1983; Meredith, 1988; Highstein et al., 1992; McCormick and Braford, 1994; Straka and Baker, 2011; McCormick and Wallace, 2012). The peripherally located epithelial endorgans for these four sensory modalities develop from a common placodal region and the sensory transduction mechanism is largely based on the same ancestral hair cell-type receptor (Fritzsch et al., 2002; Modrell et al., 2011). This ubiquitous mechanoreceptor-type cell is used to detect and convert respective physical forces into neuronal activity clearly distinguishing between electro- and mechano-sensory stimuli. Peripheral octavolateral hair cells are linked to first order afferent nerve fibers from either the lateral line/electrosensory neuromasts on the body surface or specialized vestibular/auditory endorgans to distinct nuclear regions in the dorsal hindbrain (McCormick and Braford, 1994; McCormick and Hernandez, 1996; Straka and Baker, 2011).

**FIGURE 2:**
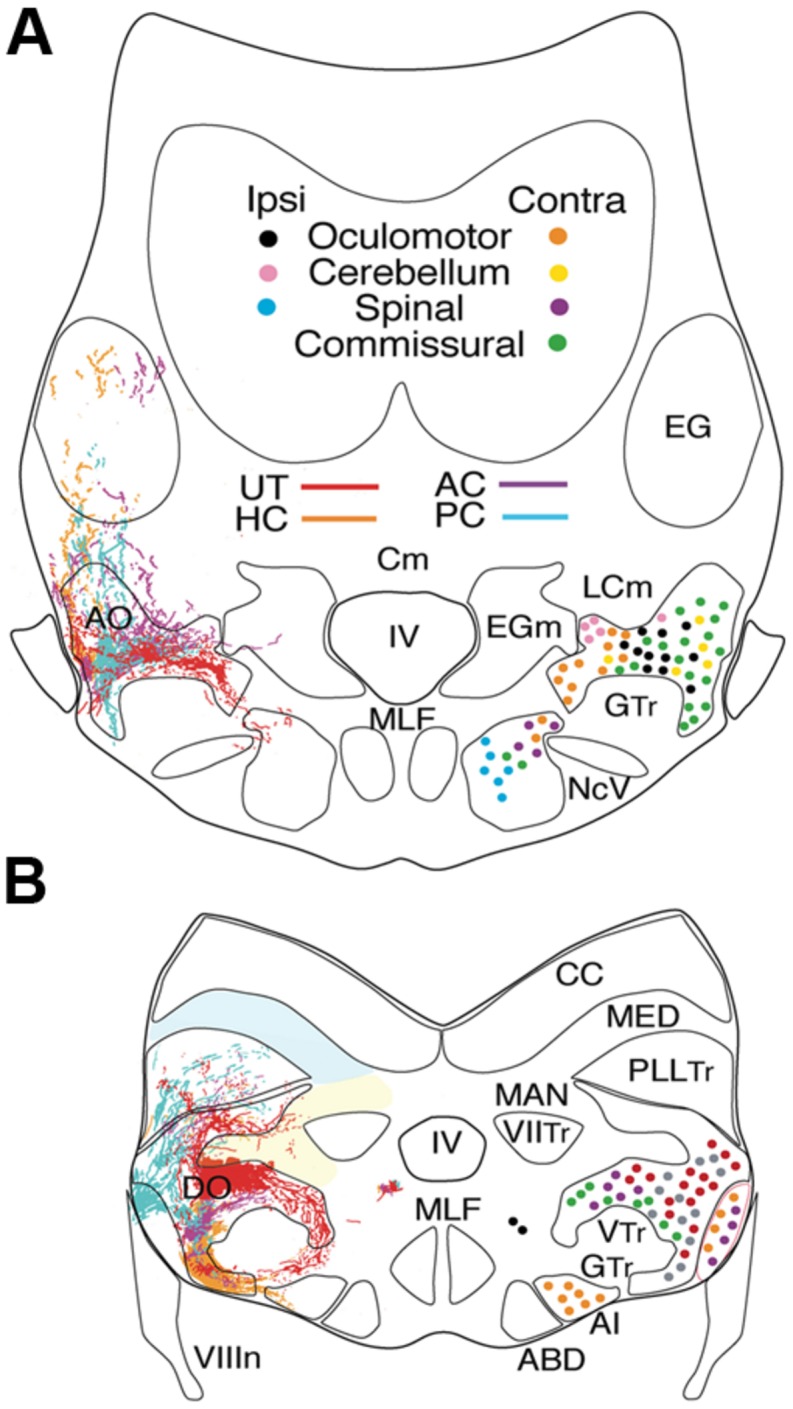
**Afferent and efferent vestibular pathways in adult goldfish.** Schematic cross sections at the level of r3 **(A)** and r5 **(B)** showing first order canal and utricular afferent terminations. Efferent projections to ipsi- and contralateral extraocular, spinal, cerebellar, and vestibular targets are color-coded. Reconstruction based on own data (Straka and Baker, unpublished) and published material (McCormick and Braford, 1994). AI, abducens internuclear neurons; ABD, abducens motoneurons; AO, DO, anterior, descending octavolateral nucleus; CC, cerebellar crest; EG, eminentia granularis; LC, caudal lobe; MAN, medial auditory nucleus; MED, lateral line medial nucleus; MLF, medial longitudinal fasciclus; T, tangential nucleus; GTr, secondary gustatory tract; PllTr, posterior lateral line tract; VTr, descending tract of the Vth cranial nerve; VIITr, central tract of the seventh cranial nerve.

Vestibular sensory function in the somewhat inappropriately called “inner ear” of early vertebrates consists of separate semicircular canals attached to a single sac-like structure, the utricle with additional sac-like structures, the saccule, and the lagena, utilized for audition and/or graviception (Fritzsch et al., 2002) and magnetoreception (Wu and Dickman, 2012). The semicircular canals are oriented at right angles from each other comprising different planes. Expansions in each canal known as ampullae contain a crista covered by a gelatinous cupula that is deformed by fluid motion during rotational movements of the head/body, thus acting as force transducers of angular acceleration. By contrast, the utricle is filled with movable carbonate stones overlying fixed hair cells and is highly effective for transducing linear acceleration. The major role of the utricle in all vertebrates is for sensing inertia through detection of static changes in head/body position relative to the earth gravitation vector thereby creating an internal “frame of reference” for orientation and locomotor behaviors (Baker, 1998; Hess and Angelaki, 1999). Vestibular afferent fibers are segregated from all other eighth nerve components and project to the most lateral neuronal subgroups in the hindbrain (**Figures 2A,B**). Based on their evolutionary-conserved neuronal projections to brainstem and spinal regions responsible for the control of balance and locomotion, the lateral portions of the octavolateral nuclei can be clearly distinguished from the more medial, and dorsal nuclei that process acoustic and lateral line sensory signals (**Figures 2A,B**).

## HINDBRAIN SEGMENTAL ORGANIZATION OF VESTIBULAR SUBGROUPS

The most comprehensive developmental and topographical mapping of overall vestibular organization has been carried out in cyprinids (goldfish and zebrafish). Based on the cellular organization, the vestibular nuclei are generally illustrated as five major subdivisions of second order neurons related to balance, postural control, and motion (**Figure 1B**). Each subdivision can be further divided into distinct functional groups involved in the control of eye and body motion based on (1) regionally restricted terminations of first order afferent fibers from the different peripheral vestibular endorgans, (2) anatomical tract tracing from efferent projection areas in the midbrain, hindbrain and spinal cord (SC), and (3) electrophysiological correlates of second order neurons during different behavioral paradigms (Highstein et al., 1992; McCormick and Braford, 1994; McCormick and Hernandez, 1996; Graf et al., 2002; McCormick and Wallace, 2012). The structural and functional similarity of the major vestibular cell groups across different fish species allows vestibuloocular, vestibulospinal, vestibulocommissural, and vestibulocerebellar neurons to be identified as distinct populations (**Figures 2A,B**).

**FIGURE 3:**
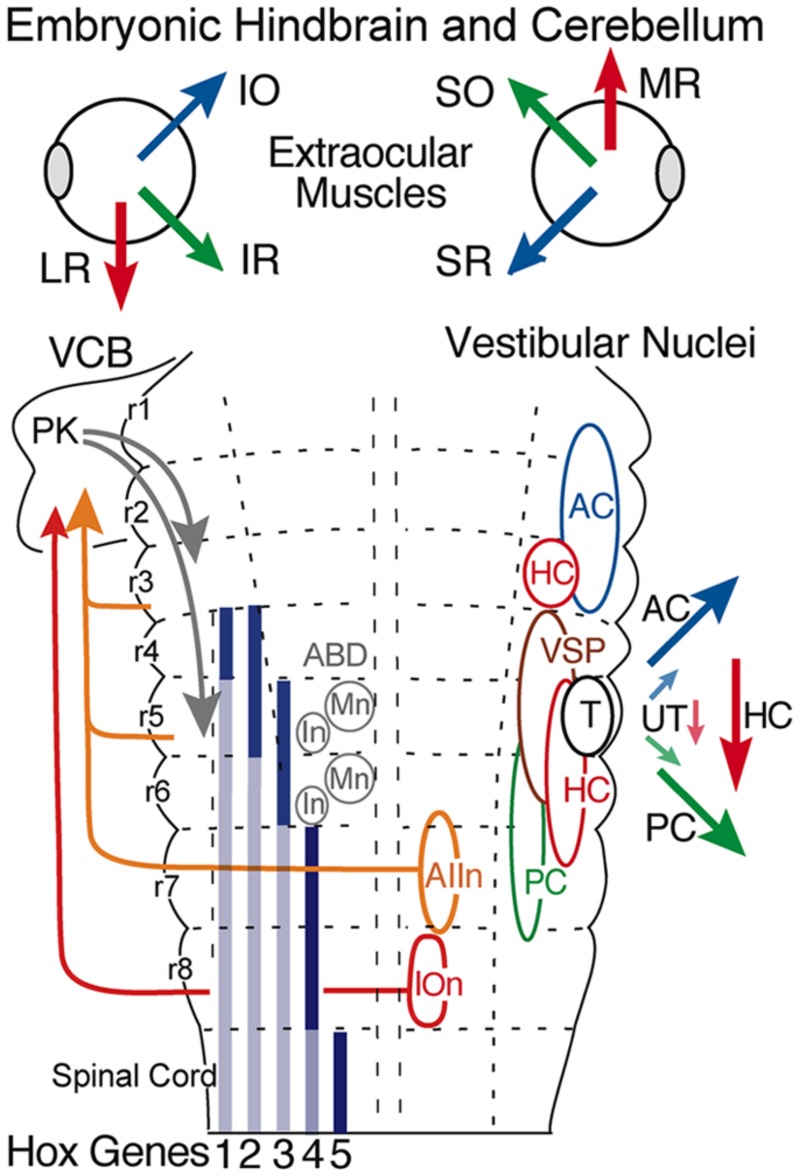
**Hindbrain segmental organization of vestibular nuclei with schematic diagram of canal and otolith-specific vestibuloocular behaviors and hox gene expression.** Corresponding color-coded arrows and excitatory vestibuloocular subgroups in r1-3 and r5-7 indicate the close spatial alignment between canal planes and vertical, oblique, and horizontal extraocular muscle pulling directions. Afferents from the inferior olive (r8) and vestibular subgroups (r3, r5, r7) to the vestibulocerebellum (VCB) are necessary for vestibuloocular reflex plasticity. Adapted from Straka et al. (2001). ABD, abducens nucleus; AC, PC, HC, anterior vertical, posterior vertical, horizontal canal; In, internuclear neuron; IOn, inferior olivary neurons; IO, SO, inferior, superior oblique; IR, LR, MR, SR, inferior, lateral, medial, superior rectus; Mn, motoneuron; PK, Purkinje cell; T, tangential nucleus; UT, utricle; VSP, vestibulospinal.

**FIGURE 4:**
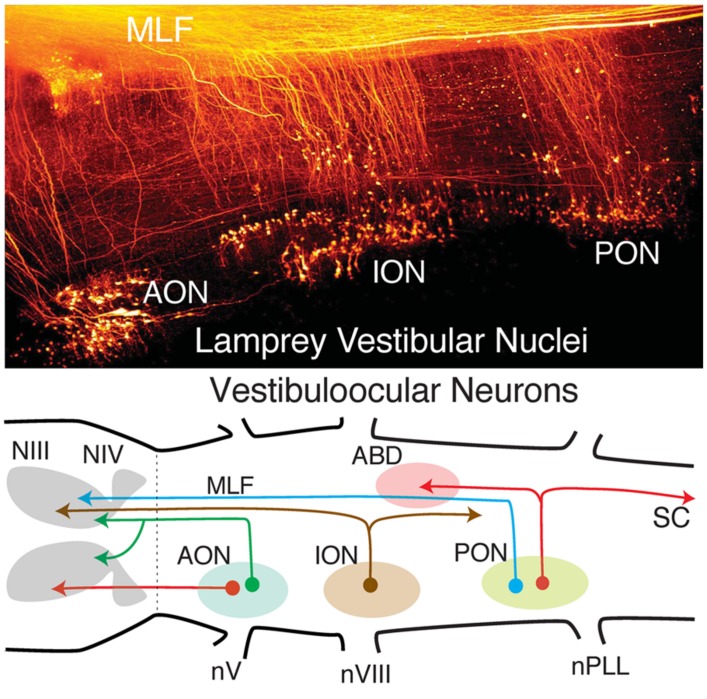
**Organization of lamprey octavolateral vestibular nuclei.** Confocal image stack (top) showing retrogradely labeled vestibular neurons in three midbrain-projecting nuclei. Schematic diagram (bottom) of crossed and uncrossed vestibuloocular neurons in the anterior, inferior, posterior octavolateral nuclei (AON, ION, PON) projecting to motoneurons in the oculomotor (III), trochlear (IV), and abducens (ABD) nucleus. PON neurons (red lines) project to ABD Mns and the spinal cord (SC). Organizational scheme based on data reported by Gilland and Baker (1995) and Pombal et al. (1996). nV, trigeminal nerve; nPLL, posterior lateral line nerve; nVIII, vestibular nerve; MLF, medial longitudinal fasciculus.

**FIGURE 5:**
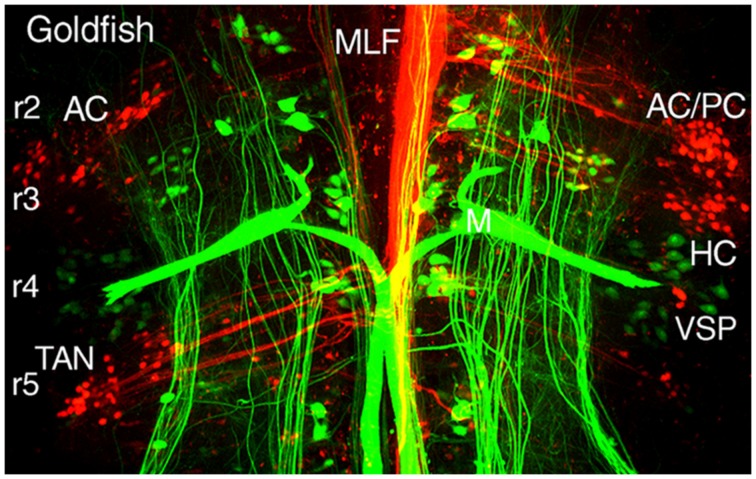
**Segmental organizations of vestibuloocular, vestibulospinal, and reticulospinal neurons in a young adult goldfish.** A confocal image stack illustrates arrangements of canal-specific oculomotor/trochlear (red) in r2 (AC, PC), r3 (HC), r5 (TAN), and spinal cord-projecting neurons (green) in r4-5 adjacent to the large Mauthner cells in r4. Adapted from Suwa et al. (1996).

All vestibular subgroups develop within a hindbrain segmental framework that consists of eight well-defined neuroepithelial segments, rhombomeres (r) 1-8 (**Figure 3**; Vaage, 1969; Gilland and Baker, 1993; Straka et al., 2001; Gilland and Baker, 2005). The vestibular column originates largely from embryonic compartments r2-7, and thus spans nearly the entire rostro-caudal extent of the hindbrain (**Figure 3**). Distinct subsets of vestibuloocular neurons in r1-3 and r5-6 and of vestibulospinal neurons in r4-6 occupy unique segmental compartments, respectively (**Figures 3–5**). Each hindbrain segment is characterized by a combinatorial expression of distinct families of transcription factors that play a dominant patterning role as, e.g., Hox1-5 genes (**Figure 3**; see e.g. Davis and Stellwag, 2010). Each of the segmentally restricted vestibular subgroups is therefore defined by an evolutionary conserved set of spatially restricted activation of regulatory genes (Baker, 1998). Thus, the location of specific vestibular subgroups along the hindbrain segmental axis is not determined by either a random translocation or migration of stem cells from a common germinal epithelium, but by a genetically defined blueprint for each vestibular functional phenotype. This genetic and compartmental blueprint appears to be shared between all species of fish. Based on knowledge of neuronal position within the embryonic hindbrain, it is therefore possible to recognize the ontogenetic origin of each vestibular subnucleus in adult fish with respect to the underlying hindbrain segmental scaffold and to link it with the adult architecture of the various vestibular subdivisions (Baker, 1998; Fritzsch, 1998).

## EVOLUTION OF VESTIBULAR SUBGROUPS

Vestibular neuronal subgroups evolved long before fishes in the earliest jawless vertebrates as, e.g., lamprey (**Figure 4**; Gilland and Baker, 1993; Pombal et al., 1996; Baker, 1998). Interestingly, lampreys have only two semicircular canals, the anterior (AC), and posterior vertical canal (PC), that contribute equally to detection of horizontal components of angular acceleration (Fritzsch et al., 2002). Appropriately directed eye movements in lamprey are produced for angular rotations about all three axes, including horizontal vestibuloocular reflexes from neurons located in the well-delineated rostral (AON) and caudal (PON) subgroups (**Figure 4**). The vestibular subdivision of the octavolateral nuclei was modified in jawed fishes after a third canal, the horizontal (HC), was added to the ontogenetic blueprint. Notably, the structural redesign in vestibular subgroups, in particular the unique central horizontal eye movement circuitry, has been conserved ever since it evolved in fishes to present day mammals (**Figure 3**; Fritzsch, 1998).

The fundamental hindbrain vestibular blueprint in lampreys consists of rostral, anterior (AON), and caudal, posterior (PON) semicircular canal-related subdivisions separated by an intermediate (ION) otolith-related group of neurons (Pombal et al., 1996; Straka and Baker, 2011). However, central vestibular subgroups were formed even earlier in the primitive jawless hagfish from a single canal and otolith acting as directional angular and linear accelerometers. Thus, the anterior to posterior distributions of second order neurons/nuclei from r2-7 likely evolved from an expansion of the initial gravitoinertial intermediate subgroup that arose directly adjacent to the eighth nerve entry in r4 (**Figure 3**). Accordingly, anterior semicircular canal-related neuronal subgroups along with the first major cerebellar nucleus (i.e., vestibular), originate from r1-3 (**Figure 3**). The vestibulocerebellum (VCB) is therefore the phylogenetically oldest part of the cerebellum. This embryological blueprint schematically illustrates the vestibulocerebellar circuitry showing the closely linked evolutionary history of neuronal processing co-adapted for the control of eye and body motion in a three dimensional environment that was little modified during the subsequent transition of vertebrates from water to land (Baker, 1998; Fritzsch, 1998; Straka et al., 2006).

## EVOLUTION OF SENSORY AND MOTOR FUNCTION

**FIGURE 6:**
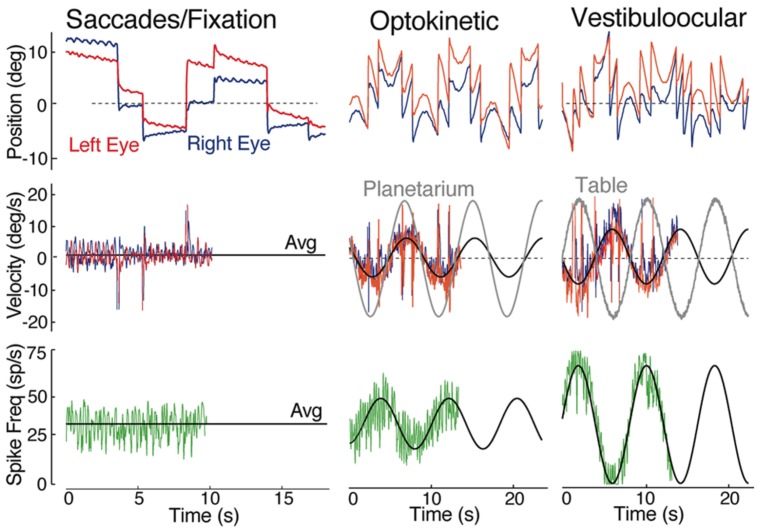
**Spontaneous, visual and vestibular-evoked horizontal eye movements in adult goldfish.** Eye position (top row) and eye velocity (middle row) of the right (blue trace) and left eye (red trace) with the correlated discharge of a vestibular neuron (bottom row) during spontaneous saccades with periods of fixation, horizontal sinusoidal optokinetic stimulation (planetarium), and vertical axis sinusoidal head rotation (vestibuloocular); the black line indicates the average (Avg) eye velocity (middle row); and neuronal firing rate. Data were adapted from Green et al. (1997).

Since the earliest jawless vertebrates, the vestibular nuclei appear to align with the location of the peripheral anterior and posterior semicircular canals surrounding a central otolith (**Figures 3 and 4**). In addition, first order vestibular afferents likely exhibited the ability to contact neurons in any of the r2-7 derived subnuclei. As a result, in the most primitive jawless and jawed fishes the vestibular system was quickly converted from point-to-point, hard-wired canal/otolith specific subgroups relaying purely sensory signals to remarkably plastic subsets of neurons that effectively could both generate and relay on-line motor signals as illustrated by the specific visuo-vestibular behavioral repertoire of horizontal eye movement signals in goldfish second order neurons (**Figure 6**; Dichgans et al., 1973; Green et al., 1997). This afferent convergence also provided the basis for a species-specific structural and functional plasticity of second order neuronal processing mediated by the newly acquired cerebellar circuitry (**Figure 3**).

From a morpho-physiological perspective, interconnections between the bilateral vestibular nuclei may have had the most significant effect for vestibular function (Straka and Dieringer, 2004). A midline crossing of vestibular axons likely originated in jawless vertebrates as a means to provide axis-specific canal feed forward interactions between vestibular subgroups controlling, for example, agonist/antagonist eye muscles in each eye (**Figure 3**). The hindbrain commissural innovations appeared to evolve largely, if not exclusively, in the direction of anterior canal second order inhibitory neurons contacting contralateral (plane-specific) posterior canal-related inhibitory and excitatory vestibular neurons. This arrangement had a significant impact on subsequent evolution of vestibulocerebellar circuitry in which, for example, efferent neurons (Purkinje cells in **Figure 3**), only terminate directly onto either excitatory or inhibitory second order neurons originally associated with the anterior canal system (Baker et al., 1972; Ito et al., 1973). Interestingly, this exotic synaptic arrangement is maintained in the classical three-neuronal vestibuloocular reflexes from fish through mammals (Straka and Dieringer, 2004). Consequently, vestibular and cerebellar circuitry in fish largely arose from an ancestral anterior/posterior semicircular canal second order vestibular neuron blueprint that includes a subsequent, phylogenetically derived, horizontal semicircular canal pathway (**Figure 3**). While the asymmetry of the fish pathways appears more complicated than necessary, especially from the perspective of just aligning paired canals across the midline (AC-PC, PC-AC, or HC-HC), evolution of inhibitory canal-specific connections across the midline was a parsimonious solution to both structural and functional constraints imposed by an original two canal sensory system that endured for around 100 million years.

## CENTRAL TERMINATIONS OF SEMICIRCULAR CANAL AND UTRICULAR AFFERENTS

The distribution of first order afferent fibers from individual canals (AC-PC-HC) and the utricle allows central vestibular nuclei to be recognized in respect to head/body motion. Anterior and posterior semicircular canal afferents in fish terminate predominantly, even though not exclusively, in the anterior octavolateral nucleus (AO) that originates from r1-3 and in the r5 tangential nucleus (T) (**Figure 2**; McCormick and Braford, 1994; Tomchik and Lu, 2005; Maruska and Tricas, 2009). Afferent projections from these two canals are less dense in the descending octavolateral nucleus (DO) that originates from r5-6 and the r7-8 posterior octavolateral nucleus (PO; **Figures 2** and **3**). In contrast, the horizontal canal afferent termination coincides with the parvocellular part of the magnocellular octavolateral nucleus (MO) that originates from r3-4 as well as more uniformly throughout DO and T (**Figures 2, 3**).

First order utricular afferents terminate predominantly in MO, T, and DO, thus overlapping to a considerable extent with those from the horizontal canal (**Figure 2**); however they also extend ventromedially outside the octavolateral nuclei toward the horizontal eye movement nuclei (ABD and AI; Straka et al., 2006). Significant for all sensorimotor behaviors in fish, there are virtually no vestibular nuclei (from r2-7) that receive exclusive afferent projections from a single peripheral endorgan. Rather, first order afferents overlap more or less completely, even though “hot spots” exist in some nuclei (**Figure 2**). This wide spread vestibular pattern is thus clearly different from topological maps of other sensory systems such as the closely related visual system. The absence of a sensory point-to-point map is consistent with the finding that signal processing in central vestibular nuclei is more spatially organized according to motor targets rather than to topological hindbrain location (Straka and Dieringer, 2004; Straka, 2010). These observations are, in turn, quite compatible with the demonstrated roles of the vestibular system in sensorimotor transformations contributing to the control of eye position and posture.

## HINDBRAIN VESTIBULOOCULAR ORIGINS AND PROJECTIONS

Perhaps the chief structural attribute of the vestibular system is the spatially precise projection to motoneurons located in the oculomotor, trochlear, and abducens nuclei that produce compensatory, three-dimensional eye movements in all species of fish (Graf and Baker, 1985a,b; Suwa et al., 1996, 1999; Graf et al., 1997, 2002). The large medial longitudinal fasciculus pathways (MLF; **Figures 4**, **5**, and **8**) formed by vestibuloocular neurons illustrate the essential link that evolved between sensory and extraocular reference frames that could in many early vertebrate species nearly perfectly stabilize gaze in response to either retinal image drift or eye/head body motion (**Figures 3 and 9**). The vestibular neuronal subgroups critical for sensorimotor transformation of linear and angular acceleration signals are located laterally within the borders of r1-3 and r5-7 (Suwa et al., 1996; Gilland et al., 2004). The principle for the underlying connections is simply that of matching spatial vector orientations for vertical and oblique vestibuloocular reflexes that stabilize vertical and torsional eye movements during body movements around the roll and pitch axis (**Figures 3**, **8**, and **9**). Afferent input from the two vertical semicircular canals comprise the major input for vestibular subgroups activating extraocular muscles (SR, IR, SO, IO) with corresponding spatially aligned pulling directions (**Figure 9**, e.g., goldfish and flatfish; Graf and Baker, 1985a,b, Graf et al., 1997).

## VERTICAL AND TORSIONAL VESTIBULOOCULAR NEURONS

Different populations of second order vestibular neurons form spatially, non-overlapping subgroups within the AO in which neuronal phenotypes can be functionally distinguished with respect to their respective extraocular motor target (Baker, 1998; Fritzsch, 1998; Gilland et al., 2004). Oculomotor/trochlear-projecting vestibuloocular neurons in the AO subdivide into three populations: a somewhat dispersed cell group excites contralateral SR and IO motoneurons and two densely clustered populations inhibit ipsilateral SO, IO, SR, and IR motoneurons (**Figures 2A** and **3**). The subgroup of vestibular neurons that excites contralateral IR and SO motoneurons is located more caudally in the DO/PO (**Figure 2B**). The caudal r5-7 position of these neurons is expected from the observed PON location in jawless fish and lamprey (**Figure 4**). Axons from both the rostral and caudal vestibuloocular subgroups reach their respective extraocular motor subdivisions in the oculomotor/trochlear nuclei by axonal pathways in the ipsi- or contralateral MLF (**Figure 5**).

## HORIZONTAL VESTIBULOOCULAR NEURONS

**FIGURE 7:**
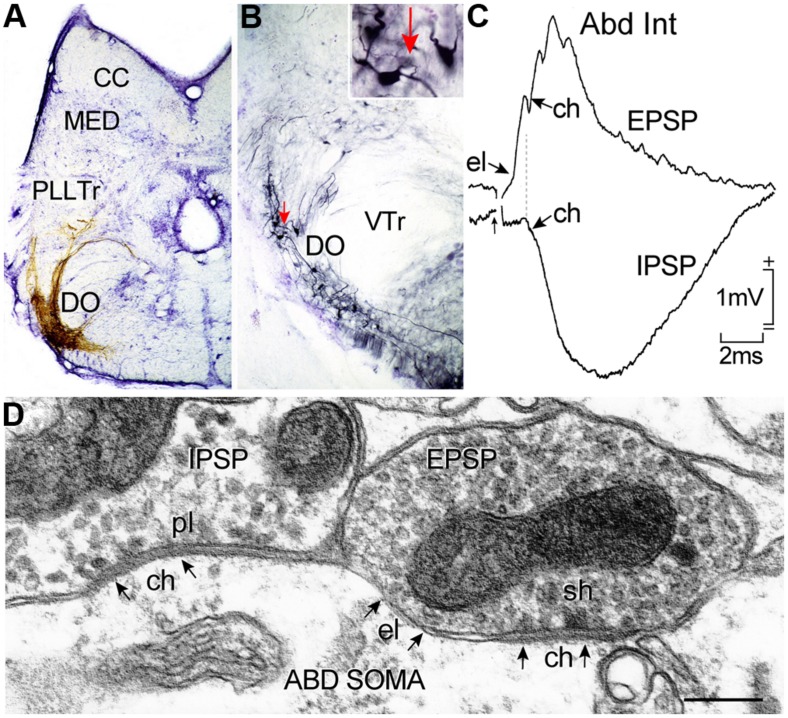
**Horizontal canal termination, second order vestibular neuron location and morpho-physiological correlates of excitatory and inhibitory vestibular inputs to abducens motoneurons and AI neurons.** Nissl-stained transverse section through a goldfish hindbrain illustrating biocytin-labeled horizontal canal afferent terminations **(A)** and vestibular neurons in the DO **(B)** that were retrogradely labeled from the contralateral ABD nucleus; a labeled neuron (red arrow) in **(B)** is shown at higher magnification in the inset. Crossed EPSP and uncrossed IPSP in an abducens internuclear neuron following electrical stimulation of the horizontal semicircular canal nerve on both sides, respectively **(C)**; responses are mediated by electrical (el); and/or chemical synapses (ch) between inhibitory (pl) and excitatory vestibular (sh) axonal terminations on abducens neurons as illustrated in the electronmicroscopic image **(D)**. CC, cerebellar crest; DO, descending octavolateral nucleus; MED, lateral line medial nucleus; pl, pleiotropic; PllTr, posterior lateral line tract; sh, spheroidal; VTr, descending tract of the Vth cranial nerve; VTr, descending tract of the Vth cranial nerve. Data in **(C,D)** adapted from Graf et al. (1997) and Green et al. (1997).

The principal vestibular subgroups implicated in horizontal vestibuloocular reflexes are located in the ventral part of the DO (**Figures 2** and **7**; Green et al., 1997). Nearly mirror-symmetrical, crossed excitatory, and uncrossed inhibitory projections target four separate, ventrally located, subgroups of abducens (Abd) motoneurons, and internuclear (AI) neurons. These interdigitating motor/internuclear nuclei innervate the lateral rectus (LR) and contralateral medial rectus (MR) muscles (via MR motoneurons) to produce horizontal conjugate motion of the left and right eye (**Figure 9**; Pastor et al., 1991). AI neurons are exclusively responsible for contralateral MR adduction and along with ipsilateral LR abduction produce parallel symmetrical eye movements (**Figure 6**). However, conjugacy of eye movements in fish is much more apparent than real. Foveae are absent in the majority of fish species, and even when present would not likely produce stereoscopic vision by fusion of targets between the two eyes. Moreover, from early larval onset to adults, monocular horizontal eye movements can be elicited with suitable visual/vestibular stimuli thus demonstrating separate r5-6 populations of vestibular neurons to selectively target either Abd or AI neurons (Debowy and Baker, 2011). Nonetheless, irrespective of whether a particular inhibitory or excitatory r5-6 vestibular neuron projects to Abd motoneurons or AI neurons, the neuronal signature is always a combined head/eye velocity signal (**Figures 6** and **9**). This neuronal correlate is observed during either head rotation or optokinetic visual stimulation about the vertical axis. By contrast, the r5-6 vestibular neurons do not exhibit any activity during scanning horizontal eye motion (**Figure 6**), suggesting a different embryonic origin and evolution of the hindbrain saccadic/fixation system (Baker and Gilland, 1996).

Interestingly, a subgroup of horizontal head velocity neurons is located rostrally in r3 and projects exclusively to ipsilateral MR motoneurons (**Figure 9**). The separate vestibular sites in r3 and r5-6 likely arose during the speciation event that gave rise to the horizontal semicircular canal during the transition from jawless to jawed fish (**Figures 3** and **9**). While the head velocity signal on the two subpopulations might be considered as redundant, it likely is not, as the r3 neurons subserve an essential high frequency vestibular role not provided by AI neurons. Moreover, in contrast to the r5-6 excitatory vestibular neurons projecting to Abd motoneurons, cerebellar Purkinje cells, according to their phylogenetic AON linkage in lamprey, directly inhibit the r3 vestibular neurons that project to MR motoneurons (**Figure 4**). The r3 vestibular subgroup is maintained through mammals giving rise to a well-recognized pathway (Ascending Tract of Deiter’s) to the oculomotor nucleus. The highly conserved locations of horizontal semicircular canal related second order vestibuloocular neurons within the vertebrate hindbrain clearly indicate an organizational principle centered on motor target rather than topography of spatially segregated inputs from the sensory periphery (Straka et al., 2003).

## TANGENTIAL NUCLEUS

**FIGURE 8:**
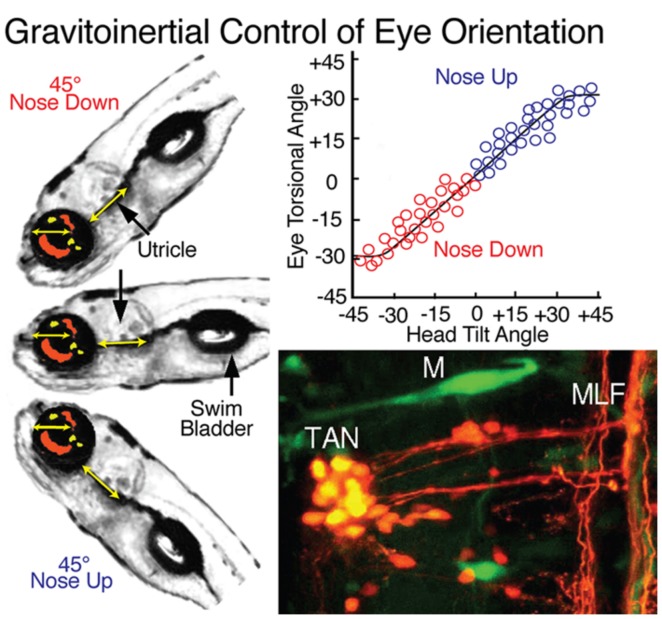
**Gravitoinertial control of eye orientation in larval zebrafish.** Compensatory static eye positions attained during 45^∘^ nose up and -45^∘^ nose down pitch are illustrated by the fish insets on the left. Precise counter-rotation of the eyes (top right) derived solely from sensorimotor transformation of utricular signals in tangential (TAN) neurons (bottom right). M, Mauthner cell; MLF, medial longitudinal fascicle. Figure summarizes results from Moorman et al. (1999); Suwa et al. (1999) and Bianco et al. (2012).

Both vertical and horizontal canal-related vestibuloocular neurons are complemented by a homogeneous, relatively small, densely clustered subgroup that forms the tangential (T) nucleus just caudal to the eighth nerve entry (**Figures 1**, **3**, and **8**; Suwa et al., 1999; Bianco et al., 2012). The functional role of this nucleus is clearly demonstrated by the close relationship between torsional angle of the eye and head tilt (**Figure 8**) in early developing fish before semicircular canals become operational (Beck et al., 2004). This nucleus serves as a gravitoinertial center with crossed excitatory projections to the midbrain oculomotor/trochlear nuclei and the contralateral SC not only in fish but also in other vertebrates (Cox and Peusner, 1990; Díaz et al., 1998; Straka et al., 2001). Second order vestibular neurons in the T nucleus receive major inputs from the utricle as well as the three semicircular canals in a spatially specific pattern. In contrast to vestibular neurons in the semicircular canal input-dominated vestibular nuclei, tangential neurons exhibit a sustained sensitivity after small tonic changes of the head position, clearly indicating an appreciable utricular influence. The likely convergence of spatially specific afferent canal inputs with sensory signals from utricular sectors with the same spatial vector orientation in individual tangential neurons (Straka et al., 2002a) forms a structural substrate that makes this nucleus a gravitoinertial relay center for vestibuloocular and postural reflexes (Suwa et al., 1999).

## VESTIBULOSPINAL PROJECTIONS

Vestibulospinal projections control gravitoinertial-related postural reflexes through activation of spinal interneurons and motoneurons. Vestibular signals to spinal targets exhibit either low dynamics more correlated with changes in body posture or high dynamics that are involved in the initiation of fast locomotor actions. Vestibulospinal neurons are generally large and well localized within a restricted region that coincides with segmental positions derived from r4-6 (**Figure 3**; Straka et al., 2001). Large subgroups of vestibulospinal neurons are located in the MO and the dorsal part of the DO with a predominance of ipsilateral projecting neurons in r4 and rostral r5 (Suwa et al., 1996; Gilland et al., 2004). A number of vestibulospinal neurons are located in the T nucleus intermingled with those that project to the contralateral oculomotor/trochlear nuclei (Bianco et al., 2012). This observation further suggests that this nucleus serves as a gravitoinertial relay center coupling eye motion and body posture. The most caudal vestibulospinal neurons originate bilaterally from the DO with predominance for ipsilateral projections. The populations of both ipsi- and contralaterally descending vestibulospinal neurons between r4-6 are morphologically rather heterogeneous and convey semicircular canal and otolith signals. The central location of these vestibular neuronal phenotypes within the vestibular column suggests an early evolutionary appearance of gravitoinertial function utilizing utricular signals for compensating pitch and roll deviations of body position in space.

## VESTIBULAR COMMISSURAL PROJECTIONS

An innate feature of the vestibular system is the mirror-image arrangement of the sensory periphery, which allows canal and otolith endorgans to act reciprocally during head motion (Malinvaud et al., 2010). Differential detection of angular head acceleration signals is centrally reinforced by spatially specific inhibitory brainstem commissural projections that interconnect vestibular neurons with bilateral coplanar semicircular canal-related signals (e.g., ipsilateral posterior – contralateral anterior; Straka and Dieringer, 2004). This connection increases the sensitivity for detection of angular head acceleration, such that even very small rotational signals can be distinguished through the central amplification. A somewhat similar organization exists for utricular commissural connections that link second order vestibular neurons with signals from spatially aligned bilateral utricular epithelial sectors. However, these functional interconnections are more complex given the 360^∘^ response sensitivity of the utricular sensory epithelium on each side (Straka and Dieringer, 2004).

Vestibular commissural neurons, as studied in goldfish, essentially subdivide into a rostral and a caudal subgroup of dispersed small to medium-sized neurons with axons that cross the midline as distinct bundles. The rostral, and largest cluster of commissural vestibular neurons, is organized in medio-caudal and rostro-lateral cell groups in the AO throughout r1-3 (**Figure 2A**) (Gilland et al., 2004). The caudal population of vestibular commissural neurons is located throughout the dorso-ventral extent of the DO and PO in r5-7 with the majority in the r6 portion of the DO (**Figure 2B**). The most caudal subgroup is clustered within a narrow band in the ventrolateral part of the PO. Given the dense projection and termination of anterior and posterior canal afferent fibers in AO, DO, and PO regions where commissural neurons are located, these latter cell groups likely establish a “push-pull” connectivity to increase the sensitivity for detection of vertical coplanar canal signals, as demonstrated earlier with morpho-physiological and pharmacological methods in frog (Holler and Straka, 2001; Malinvaud et al., 2010) and cat (Shimazu, 1972). In contrast to the above octavolateral populations, MO neurons do not contribute to commissural projections, suggesting that primarily smaller-sized neurons with lower dynamics and linear coding properties interconnect the bilateral vestibular nuclei for controlling neuronal sensitivity and gain over a large frequency range as in other vertebrates (see Malinvaud et al., 2010).

## VESTIBULOCEREBELLAR PROJECTIONS

Based on an intimate phylogenetic linkage between the cerebellum and the octavolateral nuclei, the vestibulolateral lobe is a major efferent target for central vestibular neurons (**Figure 3**). Both first and second order vestibular projections convey angular head/body acceleration as mossy fiber input to the granule cell layer of the VCB. In turn, Purkinje cells from the caudal lobe of the VCB provide a direct output to particular vestibular subgroups that project to extraocular motor nuclei (Baker et al., 1972; Ito et al., 1973). Functionally, the vestibulocerebellar loop plays an essential role in the adaptive plasticity of gaze-stabilizing motor reflexes (Pastor et al., 1994a, 1997). The major origin of second order vestibulocerebellar projections arises from two hindbrain subdivisions. A rostral subgroup with ipsilateral projection predominance is located in the dorsal part of the AO (r2/3). The small to medium-sized neurons send axons dorsally close to the pial surface into the caudal vestibulolateral lobe. A second, relatively larger subgroup of similar sized neurons, also with ipsilateral predominance, is located ventral in the DO (r6) and the PO (r7). Axons of contralateral neurons cross the midline to join the ipsilateral pathways coursing rostrally as a broad fiber bundle. These vestibular subpopulations are contiguous with another population of cerebellar-projecting neurons located ventrolaterally in r7 (Pastor et al., 1994b; Beck et al., 2006; Straka et al., 2006) that, along with inferior olivary neurons in r8, have a common ontogenetic origin, such that all precerebellar nuclei responsible for eye/head motion originate from the caudal hindbrain (**Figure 3**).

## CONSERVATION OF FISH VESTIBULAR ORGANIZATION IN OTHER VERTEBRATES

**FIGURE 9:**
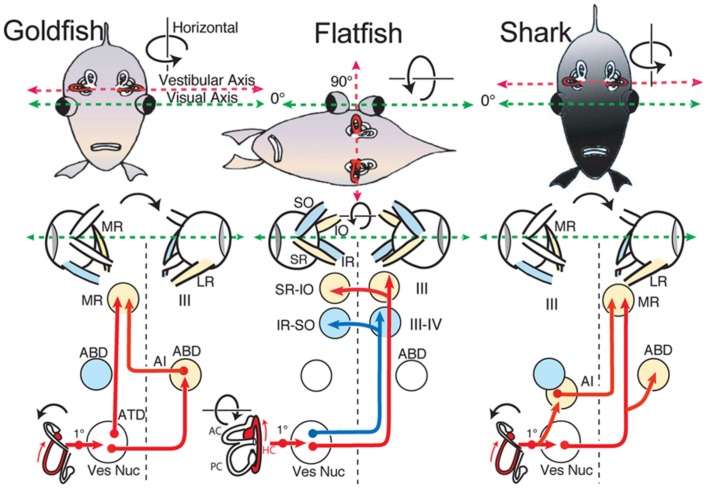
**Schematic diagram of horizontal vestibuloocular reflex circuitry in teleosts and elasmobranchs.** “Goldfish” shows the principal three-neuronal vestibuloocular reflex linking the horizontal semicircular canal with contralateral abducens (ABD) and ipsilateral MR motoneurons. ”Flatfish” shows that after 90^∘^ displacement of the vestibular relative to visual axis (metamorphosis) compensatory eye movements are produced by redirecting horizontal canal signals to vertical and oblique (SR-IO and IR-SO) motoneurons. In “Shark,” horizontal canal/second order neurons project to contralateral ABD and MR motoneurons including ipsilateral AI neurons. 1^∘^, first order vestibular neuron; ATD, Ascending Tract of Deiter’s. Schematic diagrams based on results from Graf et al. (1997; goldfish), Graf and Baker (1985a,b; flatfish), and Graf et al. (2001; shark).

Comparison of the spatial distribution of individual vestibular endorgan terminations in different species of fish, such as damselfish (Maruska and Tricas, 2009), goby (Tomchik and Lu, 2005), toadfish (Highstein et al., 1992), and more relevantly, elasmobranchs (Graf et al., 2002), reveals a vestibular pattern similar to that described herein for goldfish. Notably, central projections from comparable populations of vestibular neurons originate from corresponding subdivisions, even though details such as cell number and size differs between the various species (Straka and Baker, 2011). This conservation also includes connectivity with particular targets, even in the exceptional case of flatfish and turbot, where peripheral and/or central structural rearrangements occur during development (Graf and Baker, 1985a,b; Jansen and Enger, 1996). Young flatfish (larvae) exhibit a common fish vestibular blueprint, but vestibuloocular connections are reorganized after a body rotation along the longitudinal axis in which one eye migrates to the other side (**Figure 9**; Graf and Baker, 1983, 1990). To meet the new functional requirements for gaze-stabilizing reflexes, vertical, and oblique extraocular motoneurons in flatfish become activated in a push-pull mode by the bilateral horizontal semicircular canals (Graf et al., 2001). This structural deviation from a general conserved vestibular scheme is adapted to a particular functional need and illustrates a unique anatomical plasticity.

A comparison of vestibular system anatomy and physiology of fish with tetrapods also reveals a remarkably conserved pattern (Straka and Dieringer, 2004). In fact, afferent projections from the different vestibular endorgans in all tetrapod taxa terminate differentially in areas that correspond to those in fish. Moreover, similar subgroups of major vestibular projection neurons originate from homologous segmental positions in the hindbrain of mammals, birds, and amphibians (Glover, 1994, 2000; Díaz and Glover, 2002; Straka et al., 2001, 2002b). Even though the principle of vestibuloocular and vestibulospinal neuronal organization is similar among vertebrates, there is a remarkable difference in segmental arrangement of projection- and target-defined neurons between different taxa that led to the suggestion of a “hodological mosaic” within the vestibular nuclei (Glover, 1994, 2000). Based on this concept, neuronal location within the rostrocaudal and dorsoventral hindbrain framework would be directly linked to specification of functional phenotypes (Díaz and Glover, 2002). The overall conservation of these comparative attributes among vertebrates suggest that, from basic wiring through function, the vestibular blueprint was established quite early during vertebrate evolution and, from the viewpoint of structure more than function, has been largely conserved throughout ∼400 million years of vertebrate phylogeny.

## AUTHORS CONTRIBUTIONS

The manuscript was written and illustrated by Hans Straka and Robert Baker.

## Conflict of Interest Statement

The authors declare that the research was conducted in the absence of any commercial or financial relationships that could be construed as a potential conflict of interest.
